# River self-organisation inhibits discharge control on waterfall migration

**DOI:** 10.1038/s41598-018-20767-6

**Published:** 2018-02-05

**Authors:** Edwin R. C. Baynes, Dimitri Lague, Mikaël Attal, Aurélien Gangloff, Linda A. Kirstein, Andrew J. Dugmore

**Affiliations:** 1 School of GeoSciences, University of Edinburgh, Edinburgh, UK; 2 Université de Rennes, CNRS, Géosciences Rennes - UMR 6118, F-35000 Rennes, France

## Abstract

The action of rivers within valleys is fundamentally important in controlling landscape morphology, and how it responds to tectonic or climate change. The response of landscapes to external forcing usually results in sequential changes to river long profiles and the upstream migration of waterfalls. Currently, models of this response assume a relationship between waterfall retreat rate and drainage area at the location of the waterfall. Using an experimental study, we show that this assumption has limited application. Due to a self-regulatory response of channel geometry to higher discharge through increasing channel width, the bed shear stress at the lip of the experimental waterfall remains almost constant, so there was no observed change in the upstream retreat rate despite an order of magnitude increase in discharge. Crucially, however, the strength of the bedrock material exhibits a clear control on the magnitude of the mean retreat rate, highlighting the importance of lithology in setting the rate at which landscapes respond to external forcing. As a result existing numerical models of landscape evolution that simulate the retreat of waterfalls as a function of drainage area with a fixed erodibility constant should be re-evaluated to consider spatial heterogeneity in erodibility and channel self-organisation.

## Introduction

**Figure 1:**
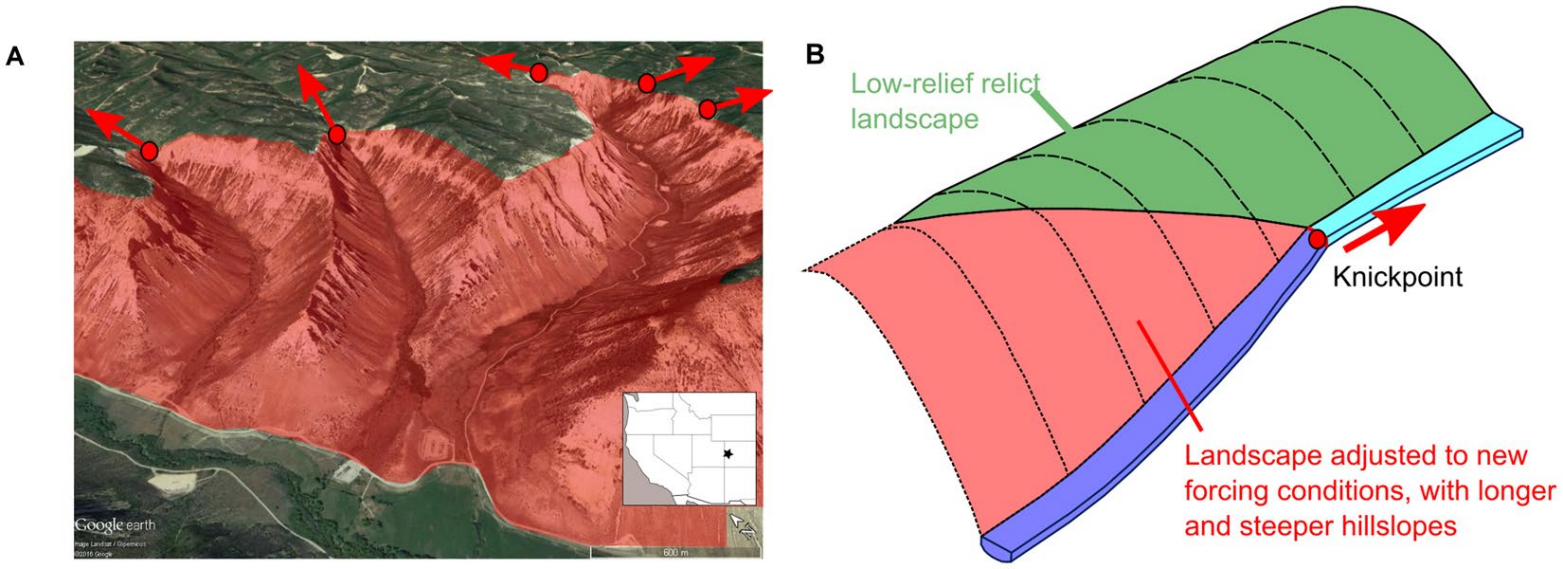
**(A)** Oblique aerial view of the Roan Plateau, Colorado, USA (image from Landsat/Copernicus in Google Earth, © Google.), with the location of knickpoints identified with the red dots. The red shaded area highlights the area of the landscape that has adjusted to the new base level conditions while the relict low relief landscape can be seen upstream of the knickpoints (Inset made with Natural Earth, Free vector and raster map data @ naturalearthdata.com). (**B)** Conceptual diagram illustrating how waterfalls or reaches of steepened channel slope, collectively termed ‘knickpoints’, represent dynamic locations within the river network, separating the part of the landscape adjusted to the new conditions (downstream – red) and those that are yet to adjust (green) (adapted from Hurst *et al*.^7^).

Understanding the processes that control how rivers adjust their elevation, location and morphology is essential for the understanding of landscape evolution across multiple spatial and temporal scales^1–3^. Regional changes in base level, such as those resulting from a drop in relative sea level or the movement of a tectonic fault, locally place rivers out of equilibrium^1–3^. Often, these perturbations appear in the landscape as abrupt vertical or near-vertical changes in channel bed elevation (i.e., a waterfall, or localised reach of increased channel slope). Here, we use the collective term ‘knickpoint’^3^ to refer to these dynamic locations. Over time, knickpoints propagate upstream, dividing a downstream reach broadly in equilibrium with its base level and an upstream reach which is yet to adjust^4,5^, typically characterised by more diffusive hillslopes with longer residence times of soils^6^, more rounded hillslopes with reduced erosion rates^7^ and broader valley bottoms^8^ (Fig. 1). Wider landscape changes propagate from the river channel after knickpoint migration, as adjoining hillslopes tend to elongate and steepen as a result of the drop in the channel elevation downstream of the knickpoint. This potentially increases the rate of hillslope sediment transport and the susceptibility to landsliding^9,10^ (Fig. 1). More generally, the phenomena of an ‘upstream incision wave’ has also been identified in other geomorphological processes such as soil erosion on hillslopes^11^, rill/gully formation^12,13^, as well as in extra-terrestrial environments including valley formation on Mars^14^. As a result, knickpoint dynamics are a fundamental aspect of landscape evolution and understanding what drives them is key.

The physics of knickpoint erosion processes are thought to depend on the mechanism of retreat such as plunge-pool erosion^15,16^, undercutting^17^ or column toppling^18,19^. Additional important factors controlling the rate at which knickpoints retreat include bedrock strength^20^, bedrock structure^21,22^, rate of sediment transport^1,22^ and the frequency of extreme flood events^19,23^. However, the complexities of the mechanisms of knickpoint retreat are often ignored in the stream power incision model, the concept that underpins much of modern quantitative geomorphology^24,25^. According to the stream power incision model, the rate of upstream knickpoint propagation is assumed to be proportional to the catchment drainage area, a proxy for river discharge:

1 $$R=K{A}^{m},$$

where *R* is the rate of knickpoint retreat, *K* is a parameter directly dependent on the bedrock erodibility coefficient, *A* is the catchment drainage area at the knickpoint and *m* is the coefficient setting the power law scaling between retreat rate and drainage area^4,5,25,26^.

This simple model has successfully predicted knickpoint retreat in the Roan Plateau, Colorado, USA^5^ (Fig. 1B), and the North Island of New Zealand^4^. However, the stream power incision model does not accurately predict knickpoint retreat in several other landscapes. These exceptions include (i) Hawai’i, where the dominant knickpoint erosion process is block plucking/toppling^21^, (ii) Scotland, where a reduction in knickpoint retreat rate has been associated with a reduction in the availability of glacial sediment for transport^22^, and (iii) Iceland, where the action of high magnitude low frequency flood events dominates the long-term evolution of the landscape^19^. In this paper, we explore the utility of the stream power incision model to predict knickpoint retreat rate in a carefully controlled experimental setup, and draw new conclusions about the morphodynamics of transient incising channels.

## Approach

Experimental modelling is a powerful tool for investigating the dynamics of the processes that drive landscape evolution^27–31^. Experimental studies using an erodible substrate of a silica cement mix have led to breakthroughs in our understanding of the processes that drive the evolution of the Earth’s surface, from the impact of tectonic uplift and climate at the orogen scale^32–34^ to the processes that control the geometry of individual channels^31^. This experimental modelling has only recently been extended to include knickpoint erosion dynamics in cohesive material^35,36^. Some investigations have considered headcut erosion in non-cohesive sediments such as sand^11,37,38^ and others have explored toppling of bedrock columns at the knickpoint face in heavily jointed bedrock in order to calibrate numerical models^18^. These studies have not, however, been specifically designed to test the assumptions that underpin the stream power incision model of bedrock incision.

**Figure 2:**
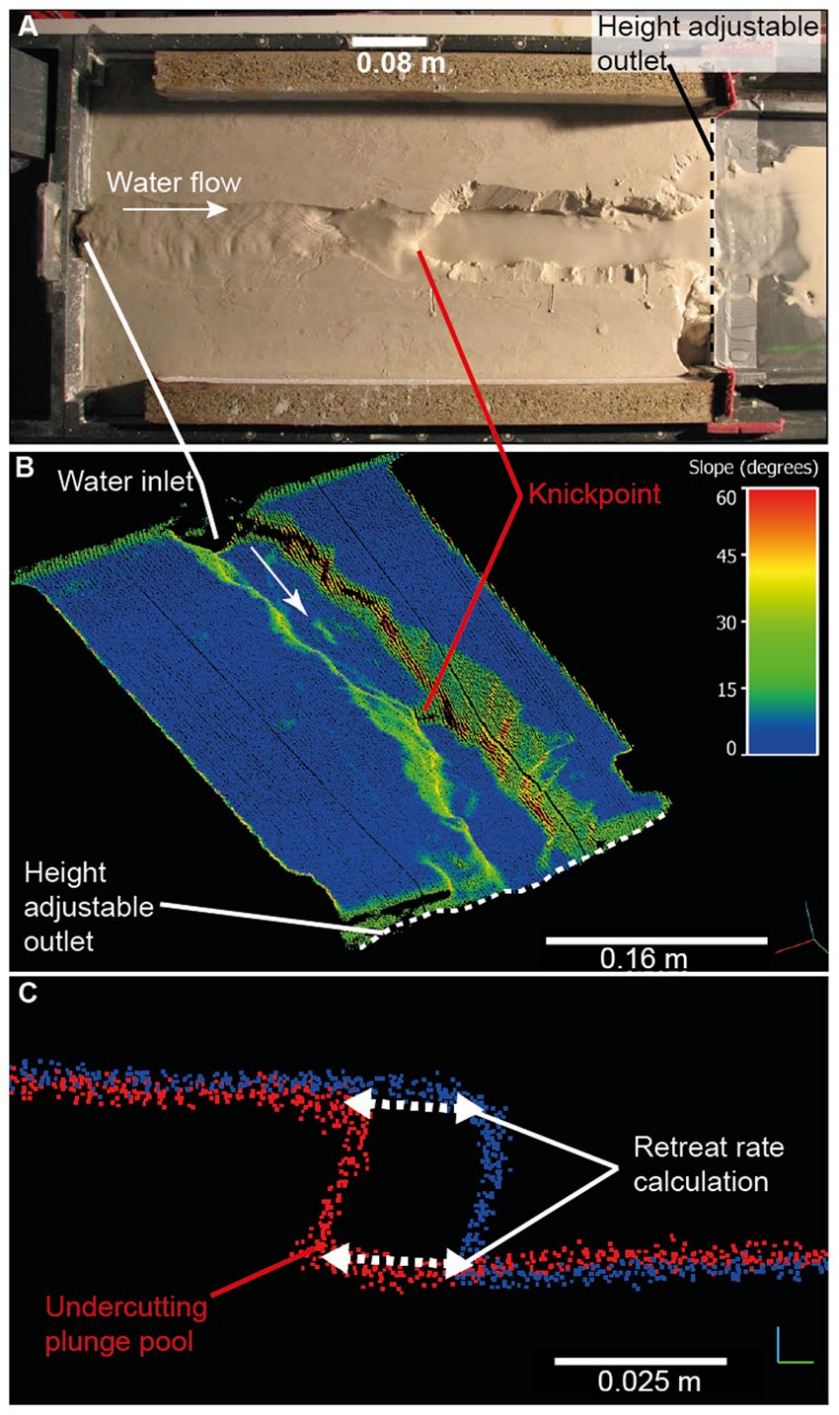
(**A**) Photograph of the 0.65 × 0.3 × 0.15 m experimental box flume, with water flowing from left to right and discharge regulated by a pump on the inflow pipe. The channel was perturbed by dropping the height-adjustable outlet to simulate instantaneous base level fall. Over the course of the experiment, the knickpoints retreated upstream (from right to left) in the form of waterfalls (some adjusted their morphology mid-experiment into steepened channel reaches) through the homogenous, cohesive, silica material, and were monitored at regular intervals using a terrestrial laser scanner. (**B**) Oblique view of an example high resolution point cloud of the channel morphology during an experiment, with colours indicating the local topographic slope. The knickpoint can be seen approximately halfway up the channel, and the white arrow indicates the flow direction. (**C**) Example of two channel profiles (extracted along the thalweg) showing knickpoints that have developed undercutting plunge pools. The dashed white arrows show the distance between the lip and the base of knickpoints in two scans at different stages of the experiment, used to calculate knickpoint retreat rate.

Here, we present results from experiments designed to quantify the role of discharge (*A*^*m*^) and relative bedrock erodibility (*K*) in setting knickpoint retreat rate and also to explore the origin of discrepancies between observed empirical data and the knickpoint retreat dynamics forecast by the stream power incision model^19,21,22^. Three mixtures of homogenous, cohesive, fine-grained silica cement were used to simulate an erodible bedrock substrate in a 0.65 × 0.3 × 0.15 m box flume fitted with an adjustable outlet that was used to generate knickpoints by instantaneous base level fall (Fig. 2A; experimental set-up described in Methodology section and SI section 1). Erosion of the silica substrate occurs through hydraulic shear by clear water rather than abrasion by transported sediment^27^. We used two types of silica grains in the mix: angular grains and spherical beads. The proportion of angular grains to spherical beads sets the relative cohesion of the material (Fig. S1), with increasing proportion of beads reducing the strength of the bonds between particles. In the experiments presented here, those where the silica mix contained 16.4% of spherical beads have the highest relative cohesion (Fig. S1).

The initial configuration of all experiments featured a channel with a vertical face 2 cm in height at the downstream edge of the flume. Experiments used discharges between 1.66–50 cm^3^/s, regulated via a pump on the inflow pipe. The width of the box flume was sufficient for a channel to form with a self-regulated channel geometry, unlike the one-dimensional experiments where width is fixed^36^. During the course of the experiments (typical duration: 60–120 minutes), the knickpoints retreated up the channel to the water inlet (Fig. 2A). High-resolution topography of the channel was surveyed using a terrestrial laser scanner (green laser that penetrates water) at 120 second intervals. This produced mm-scale quantifications of topographic characteristics, such as channel geometry and channel slope during the course of the experiment (Fig. 2B). The location of the knickpoints in the laser scans was extracted manually from the point clouds in order to measure the topographic change and calculate the retreat rate (Fig. 2C; Methods section). Hydraulic information such as water depth and shear stress was calculated using the *Floodos* 2D hydrodynamic model operating directly on a digital elevation model created from the laser scans (SI Section 2; ref.^39^). The channels were free to migrate across the width of the box, and occasionally they flowed along the side of the box for a short distance at the end of experiments; this did not affect the measurements of knickpoint retreat rate.

## Results

**Figure 3:**
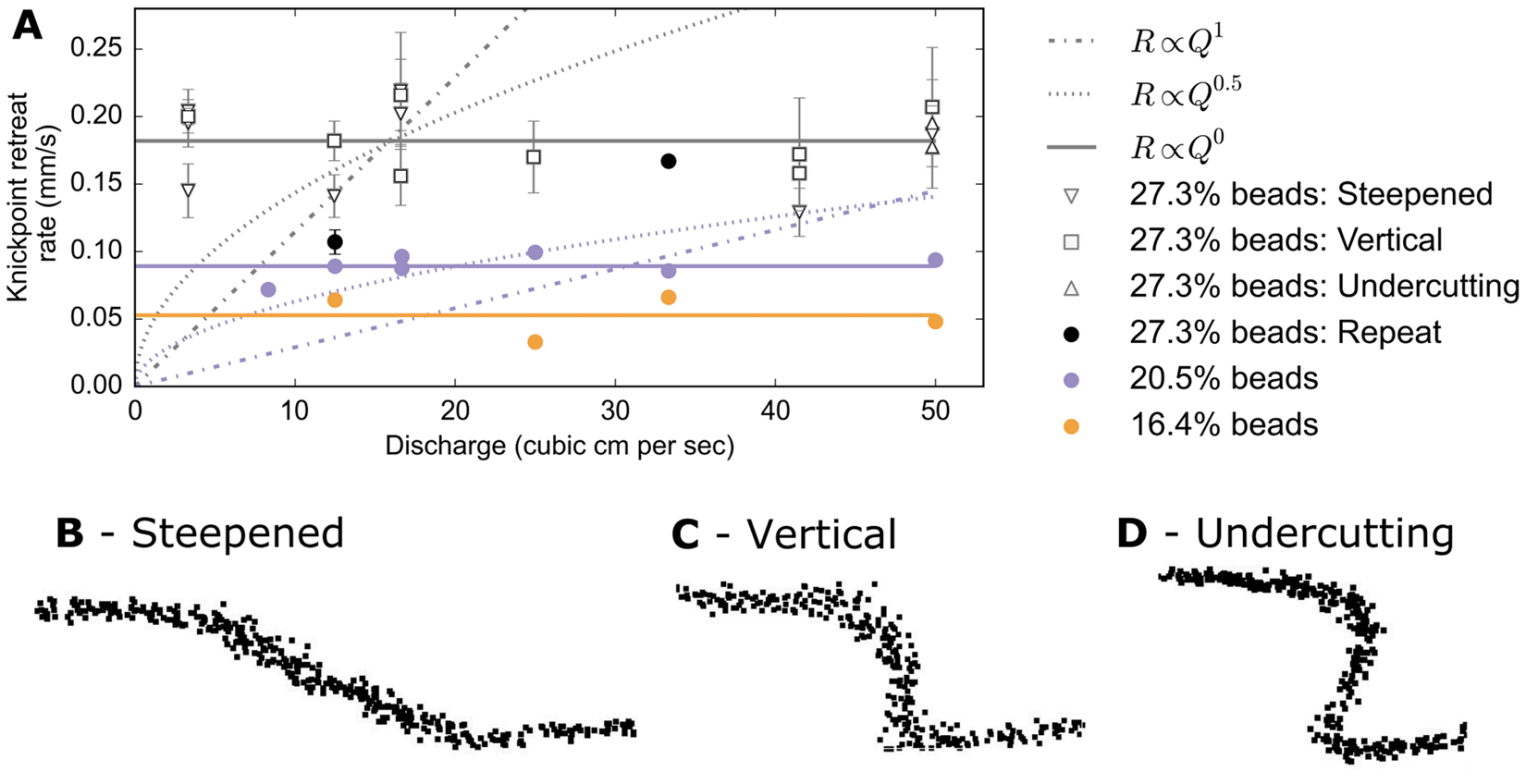
**(A)** Relationship between knickpoint retreat rate and discharge for the experiments using three different silica mixes. For the mix with 27.3% spherical beads, the knickpoint retreat rate is plotted according to the knickpoint geometry: steepened reach (circles, typical form shown in **B**), or waterfalls with either (i) vertical faces (squares, shown in **C**) or (ii) undercutting plunge pools (triangles, shown in **D**). Mean retreat rates are given for different geometries during the same experiment where a knickpoint demonstrated the same geometry for at least four consecutive point clouds (error bars corresponds to 2 standard deviations of the retreat rate). The long term retreat rate is shown for the mixes with 20.5 and 16.4% spherical beads. The relative cohesion of the paste (set by the proportion of spherical beads) has a clear control on the knickpoint retreat rate, but there is no increase in knickpoint retreat rate, despite an order of magnitude increase in discharge for experiments with the same cohesion. Superimposed on the plot are the potential relationships expected according to the stream power incision model (*R* ∝ *Q*^*0.5*^ or *R* ∝ *Q*^*1*^); the experimental data here are best described when *R* ∝ *Q*^*0*^.

**Figure 4:**
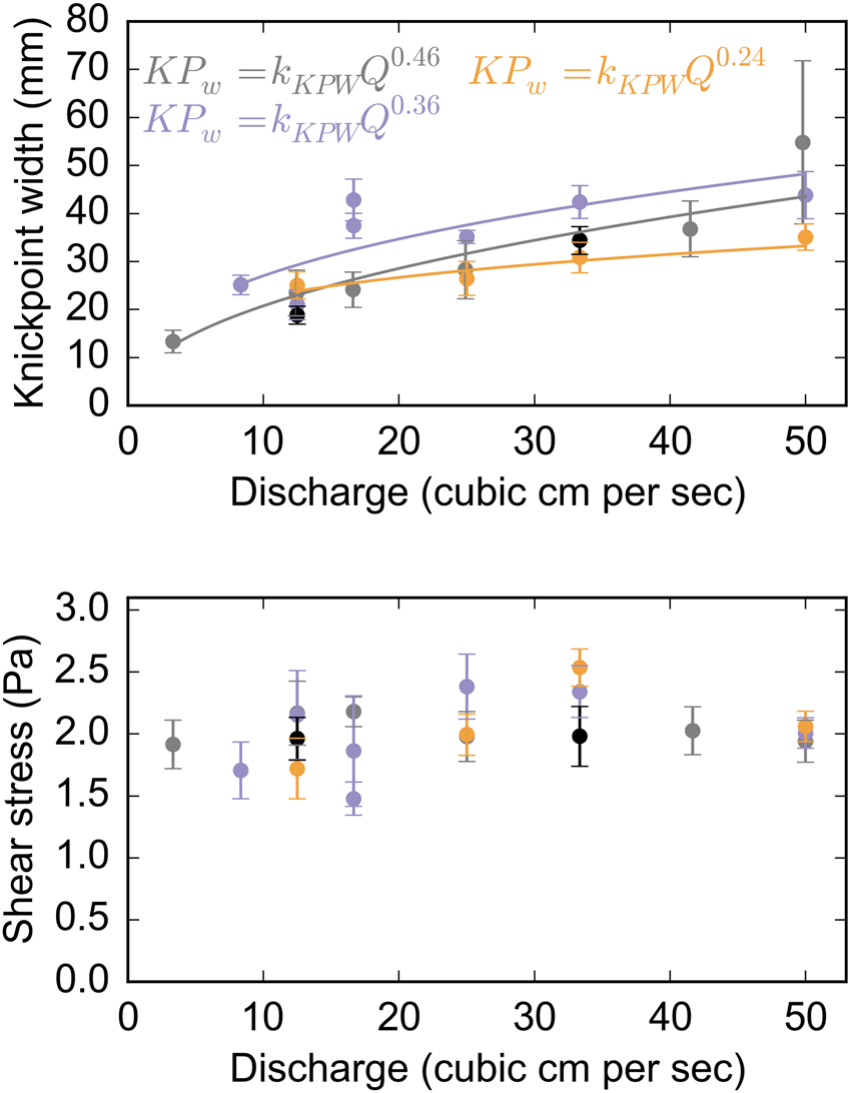
(**A**) Relationship between channel width at knickpoint lip and discharge for the experimental data. The data follow a power law such that wider channels are associated with higher discharges, with an exponent between 0.25–0.5, which is similar to those found in natural rivers^23^. (**B**) There is no clear trend between shear stress upstream of the knickpoint and discharge, suggesting the channels self-organise through variations in channel width (**A**), limiting any increase in shear stress that may drive increased knickpoint retreat rate. Error bars indicate 1 standard deviation of the shear stress values calculated for each experiment; the main source of uncertainty associated with the calculation of the water depth from the intensity of the laser return. Importantly, the shear stresses at the knickpoint lip are similar for each of the silica substrates, despite differences in the relative cohesion.

In these experiments, the main control on the knickpoint retreat rate is the relative cohesion of the substrate material (Fig. 3A), with the fastest retreat rates occurring in the silica mix with the highest proportion of spherical silica beads. The experimental datasets show that knickpoint retreat rate is insensitive to river discharge, suggesting *m* = 0 (Fig. 3A). These results do not agree with the predictions of the stream power incision model. The knickpoint retreat occurred through a combination of plunge pool undercutting, cantilever failure of the overhanging knickpoint face and hydraulic detachment on steep segments. The constant knickpoint retreat rate with increasing discharge cannot be explained by a systematic change in the knickpoint geometry. Steepened reaches, vertical faces and knickpoints with undercutting plunge pools occurred across the range of discharges, and with similar knickpoint retreat rates (Fig. 3). Increasing discharge has an impact on the geometry of the knickpoints in the experiments, with wider incised channels occurring at the knickpoint lip under higher discharges (Fig. 4A), but there is no clear associated increase in shear stress within the channel upstream of the knickpoint (Fig. 4B).

## Discussion

In this study, there is a clear link between knickpoint retreat rate and the relative strength of the bedrock material (Fig. 3A), consistent with previous field, experimental and numerical studies of knickpoint erosion^11,20,36^, bedrock channel incision^27^ and landscape evolution^40^. The strength of the bedrock substrate contributes to the value of the bedrock erodibility coefficient *K* in the stream power incision model^4^. In modelling studies, *K* is often assumed to be constant over large spatial areas despite spatial heterogeneities in the resistance of bedrock to erosion, potentially caused by lithology type^27^, or spatial patterns in chemical-weathering rates induced by climate^41^. We suggest that further consideration of variability in the bedrock erodibility coefficient should be an important component of future modelling studies, due to the potential dominant role in setting the knickpoint retreat rate.

Crucially, however, our experiments do not follow the pattern of faster knickpoint retreat associated with greater discharge, as predicted by the stream power incision model (Fig. 3A). In the experiments, erosion of the silica paste is driven by the hydraulic shear of the water detaching the particles from the bed surface. Therefore, the bed shear stress is critical in controlling the knickpoint retreat rate, but does not vary significantly between the experiments despite an order of magnitude increase in discharge. The shear stress within the channel is a function of the channel geometry (width, slope) and the discharge (SI Section 4). The experiments indicate that any increase in the shear stress driven by an increase in the discharge is offset by changes in geometry (Fig. 4A). This channel self-regulation acts to maintain the shear stress at a constant value (Fig. 4B), a similar response to the self-regulation of alluvial channel geometry that allows floods to slightly exceed the critical shear stress for bedload transport^42,43^. Due to the different relative cohesion of the silica mixes, the similar shear stresses across the experiments can explain why the mean retreat rate can be significantly faster for a given discharge during experiments with weaker material. Channel geometry (i.e., channel width) in natural rivers is expected to emerge as a balance of the local discharge, sediment supply and uplift rate^25,33,44,45^, with a timescale of adjustment as short as 10^3^–10^4^ years following changes in mean discharge or sediment composition^46^. Channel geometry can adjust to variations in discharge or sediment supply through lateral erosion of banks or vertical incision of the channel bed^47^, and we suggest that these processes lead to the self-regulation of the shear stress exerted on the bed^42,48^.

The power-law relationship between width and discharge in the experiments (Fig. 4A) is similar to the relationship between channel width and catchment drainage area in the Roan Plateau, Colorado (SI section 5), where the stream power model has been shown to accurately predict the rate of knickpoint retreat (*R* = 1.37 × 10^−8^*A*^0.54^ m/yr; ref.^5^). Recent studies have suggested that the upstream bedload sediment flux is an important driver of knickpoint dynamics^16,22,49,50^, but this is an element not included in our simple experiments. In some natural settings, the unit bedload sediment flux will increase with catchment size due to the greater sediment source area associated with larger catchments (e.g. more hillslopes connected to the channel^51^, providing more ‘tools’ for abrasion^27^). We suggest that this relationship can, in locations where erosion is dominated by abrasion, result in an apparent drainage area dependency on knickpoint retreat rate as predicted by the stream power incision model (e.g., ref.^5^). However, this relationship does not always hold, due to sediment comminution with transport distance^52^, barriers affecting sediment transport (e.g., tectonic uplift in the Da’an River, Taiwan^1^) or the exhaustion of available sediment in postglacial landscapes (e.g. Scotland^22^). From our experimental data, and the demonstrated importance of the physical processes in driving knickpoint erosion in natural settings, we suggest that patterns of knickpoint retreat are a function of the erodibility of the bedrock substrate (Fig. 2) and the complex drivers controlling abrasion or plucking/toppling. Due to the ability of channels to self-regulate the bed shear stress through their geometry, we suggest that modelling knickpoint retreat using the discharge/drainage area as a key component fails to capture the complexities of the processes driving this landscape response mechanism.

## Conclusions

Migrating knickpoints are key drivers of change within landscapes, because they are located at the dynamic boundary between a downstream reach that is in equilibrium with the new forcing conditions and an upstream reach that is yet to adjust. As such, accurate modelling of their behaviour is fundamentally important for understanding past, present and future landscape evolution. Here we demonstrate that the lithological strength is a strong control on the rate that knickpoints retreat upstream. Due to a self-regulatory mechanism of channel width to increased discharge, shear stress within the channel can remain constant, resulting in constant rates of knickpoint retreat over an order of magnitude of discharges. Thus, we suggest caution is required when using a simple scaling with drainage area or a uniform value of bedrock erodibility to interpret past knickpoint retreat as an indicator of past changes in external forcing on landscapes.

## Methods

### Experimental set-up

The experiments were carried out in the experimental modelling laboratory at Géosciences-Rennes, Université de Rennes 1, where previous microscale landscape evolution studies using silica cement have been undertaken^32–34^. The experiments presented here were carried out in a 0.65 × 0.3 × 0.15 m box flume, containing a homogenous substrate mix of 18% water, and different proportions of angular silica (D_50_ = 40 μm) and spherical silica beads (D_50_ = 40 μm). After mixing, the material was transferred into the box flume and homogenised using a high frequency concrete vibrator to re-liquefy the paste and remove any air trapped during the mixing process. The material was then left to settle for one hour before experiments began. At the end of the mixing process, the silica mixture was cohesive and water infiltration was negligible. SI section 1 contains more detail regarding the relative cohesion of the different mixes (Fig. S1), and demonstrates the relevance of the experiments to natural channels, including the demonstration of a power law relationship, with exponent 0.5, between the experimental channel width and discharge (Fig. S2).

A channel was cut (depth ~2 mm) into the top of the silica paste to focus the flow, and the slope of the flume was set to 4°. Initially, water was input into the channel at the required discharge (1.66–50 cm^3^/s, set within 2% by a regulated pump on the inflow pipe) and, in all except one set of experiments (with 27.3% beads), the water was run for enough time (>1 hour) for the channel width and slope to adjust to equilibrium conditions. After stable conditions had been reached in the equilibrium experiments, a 2 cm high vertical knickpoint was generated at the downstream end of the channel by dropping the height adjustable outlet (Fig. 2), to represent an instantaneous base level fall. After initial trials, 2 cm was chosen as an adequate balance between generating waterfall-like geometry and optimizing the number of base level drops achievable with the total volume of silica paste.

After the baselevel drop, the upstream knickpoint retreat was monitored using a terrestrial laser scanner (Leica Scanstation 2) programmed to collect a high resolution (sub millimetre) point cloud of the topography every 120 seconds. Additionally, a time lapse camera was mounted directly above the flume and collected imagery every 60 seconds. In some experiments, due to the cohesivity of the silica, the channel began to undercut the adjoining banks. This overhanging material was removed manually so the laser scanner could maintain line-of-sight with all parts of the channel bed (visible in Fig. 2A downstream of the knickpoint). In practice, this meant that no major bank collapse occurred in the channel, and the majority of the transported material comes from channel erosion at the knickpoint location. Bank collapse would only occur downstream of the knickpoints due to elevation change, so the manual removal of bank material will not have impacted the results for knickpoint retreat presented here. Data processing, extraction and analysis of the point clouds were carried out using the open source software CloudCompare 2.7^53^.

The analysis of knickpoint location and retreat rate was carried out on a subset of the point cloud data in order to accelerate the timescale of data processing. A 5 mm wide swath was extracted from the length of the thalweg in the channel for each point cloud. The location of the knickpoint lip and base was identified from each of these swaths visually, based on the inflection point of the channel slope at the upstream and downstream edges of the steepened reach. The locations of the knickpoints were compared between subsequent point clouds and the retreat rate calculated by averaging the distance between the lip and base of subsequent knickpoints and dividing it by the time between scans (extracted from the time-stamp of the point cloud collection by the laser scanner). Estimated measurement error is of the order of 1 mm, but the large number of measurements mitigate this. The consistency in retreat rates over short and long distances during each experiment suggests that the measurement error does not have a significant impact on the overall retreat rate calculations. The data analysis was performed directly on the point clouds rather than more traditional ‘DEM-of-Difference’ algorithms because some of the knickpoints developed undercutting plunge pools, a feature that would have been lost by converting the point clouds into raster layers. The knickpoint width measurements were performed at the lip of the knickpoint and the channel slope measurements were performed by calculating the change in elevation over a measured distance of ~2 channel widths upstream of the knickpoint lip. Hydraulic information (shear stress, water depth) was calculated using the *Floodos* hydrodynamic model^39^ over digital elevation models of the topography (SI Section 2).

### Data availability

The experimental data is available from E.R.C.B on request. The *Floodos* numerical model is available for download: https://osur.univ-rennes1.fr/eros/

## Electronic supplementary material

Supplementary Information

Supplementary Video

Supplementary Video
