# Supplementary material for: River self-organisation inhibits discharge control on waterfall migration
Source: Sci Rep. 2018 Feb 5;8:2444. doi: 10.1038/s41598-018-20767-6 (PMC5799191; doi:10.1038/s41598-018-20767-6)
Supplement: Supplementary file 1 — Supplementary Information [file 41598_2018_20767_MOESM1_ESM.pdf]

River self-organisation inhibits discharge control on waterfall migration.

Supplementary Information

Edwin R.C. Baynes[1,2*], Dimitri Lague[2], Mikaël Attal[1], Aurélien Gangloff[2], Linda A. Kirstein[1], Andrew J. Dugmore[1]

[1]School of GeoSciences, University of Edinburgh, UK

[2]Géosciences Rennes, UMR 6118, CNRS, Université de Rennes 1, Campus de Beaulieu, 35042 Rennes Cedex, France

*Corresponding author email address: edwin.baynes@univ-rennes1.fr

Supplementary information

SI Section 1: Material properties of the experimental silica paste, and relevance of experimental channel for natural rivers

There are two approaches to experimental modelling studies of landscape and fluvial dynamics. The first seeks to achieve direct scaling (through scaling of non-dimensional numbers such as the Froude, and ensuring that physical processes are identical) between the experimental prototype and the natural system. The second approach seeks a 'similarity of process' between the experimental prototype and the natural system by treating the experimental setup as a system in itself[1]. In the latter approach, employed in this study, the behaviour of the processes within the system are qualitatively similar to natural settings, allowing the factors that drive landscape change to be studied[2,3], although the results cannot be directly scaled up to natural rivers in space and time. In particular, processes such as flow acceleration above the knickpoint lip, plunge pool erosion, undercutting of the knickpoint face and channel banks, cantilever failure, erosion and transport of cohesive material by hydraulic shear are present in the experiments (Fig. 3C). The approach has the advantage of being able to vastly reduce the spatial and, therefore, the temporal duration of individual experiments, as well as offering a complete control on boundary conditions. While abrasion by bedload material does not occur in the experiments and would be difficult to reproduce without significantly changing the experimental design, we seize this opportunity to better highlight how knickpoint retreat in an homogeneous cohesive material occurs in response to variations in discharge and lithological strength. It is also important to note that due to the homogenous nature of the material, the impact of some natural phenomenon such as resistant hillslope material is not included within the experiments.

Following the 'similarity of process' analogue modelling approach, the cohesive silica paste used as the bedrock material in these experiments is not designed to have material properties that can be directly compared to rocks found in natural environments. However, this does not prevent the exploration of the impact of the relative cohesion of the channel substrate on the rates of fluvial processes in action in the experimental channel (i.e., knickpoint retreat). The knickpoint retreat experiments presented here were performed using three different mixes of two types of silica

material: angular silica (54.66 to 65.6% total mass) and spherical silica beads (27.33 to 16.4% total mass), with a constant proportion of water (18% total mass). The impact of the proportion of spherical silica beads on the relative cohesion of the silica paste was tested by placing a 15 x 10 x 10 cm slab of material in a tank containing 2.5 cm of water, and monitoring the time taken for the slab to collapse (Fig. S1). A higher proportion of silica beads reduces the relative cohesion of the silica mix, leading to the faster collapse of the slab (Fig. S1C). For the knickpoint experiments presented in this paper, we used the mixes with proportions of 27.3, 20.5 and 16.4%, with the 16.4% bead mix representing the strongest relative cohesion.

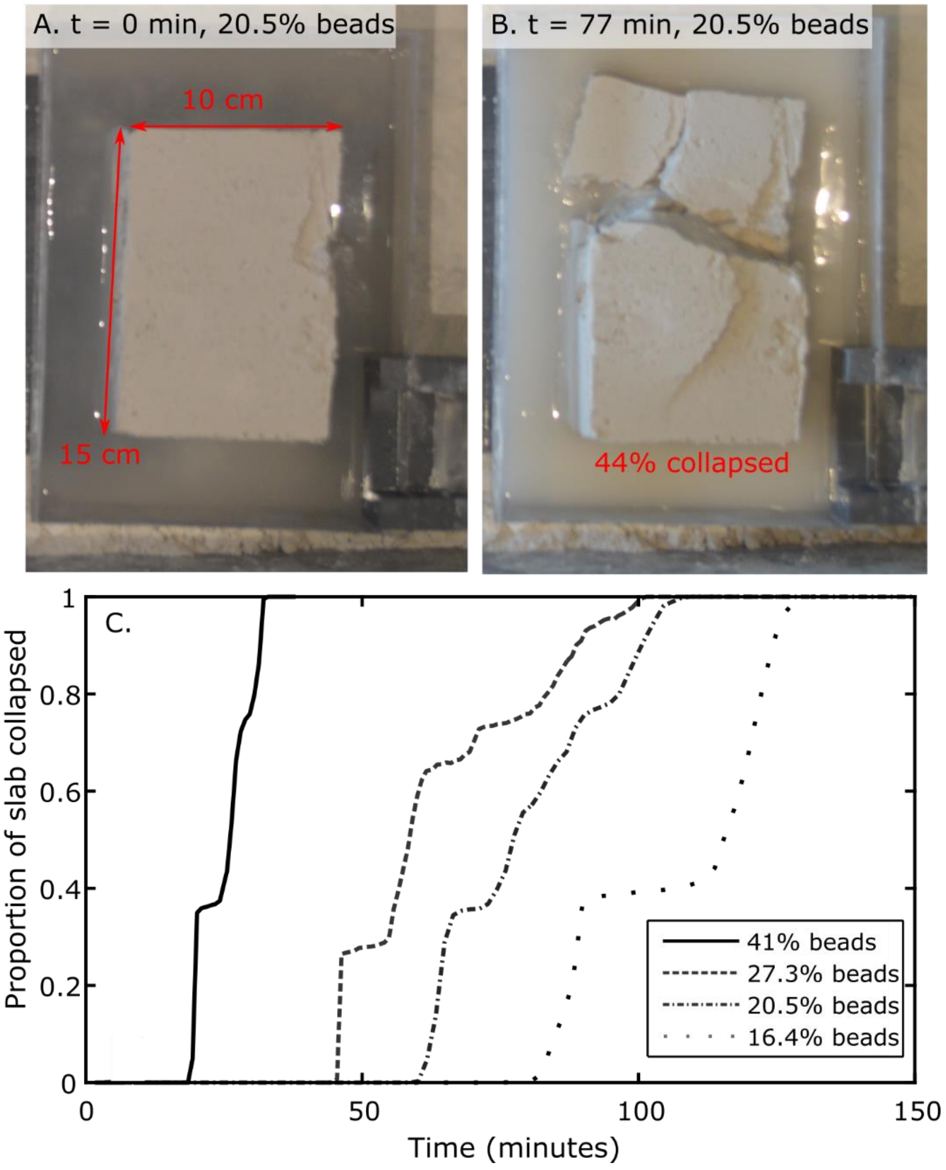

Fig. S1: **A.** Example of the slab at the start of experiment. Photo shows slab with 20.5% bead content. **B.** The 20.5% bead slab after 77 minutes, with 44% of the initial surface collapsed. The proportion of slab

collapse was calculated using Digital Elevation Models collected from laser scanner data at 2 minutes intervals. For each pixel of the DEM (pixel size = 2 mm), the pixel was defined to have ‘collapsed’ when the elevation of the pixel was 1 cm lower than its initial elevation. **C.** Time taken for a 15 x 10 x 10 cm slab of different silica mixes to collapse after being placed in 2.5 cm of water. From the experiments, the silica mixes containing a greater proportion of silica beads (e.g., 41%) started to collapse earlier, indicating a lower relative cohesion than the silica mixes containing a lower proportion of silica beads.

Beyond similarity in hydraulic processes, we note that the relationship between the channel geometry and discharge in these experimental channels is qualitatively similar to natural bedrock rivers (Fig. S2). The relationship between channel width and discharge ($W \propto Q^{\sim 0.5}$, Fig S2A) is similar to the relationship found in natural bedrock rivers (data compiled in Lague, 2014[4]). Channel slope also scales with discharge/drainage area according to a power-law relationship, with a range of values for the concavity index between 0.4-0.6 reported for ‘steady-state systems’[5]. The concavity index for the experimental channel is varied: 0.5-0.8 for the experiments at equilibrium conditions. A consistency between the concavity index in these transient experiments is not necessarily expected with the value reported for natural ‘steady-state systems’, due to adjustment of the channel geometry to new baseline conditions. The presence of the knickpoints migrating and evolving during the experiments gives an additional adjustment of the slope from ‘steady-state’ conditions. Therefore, a direct comparison between the concavity index in the experiments and natural rivers cannot be made, but the qualitative consistency suggests the experiments are a satisfactory representation of natural rivers.

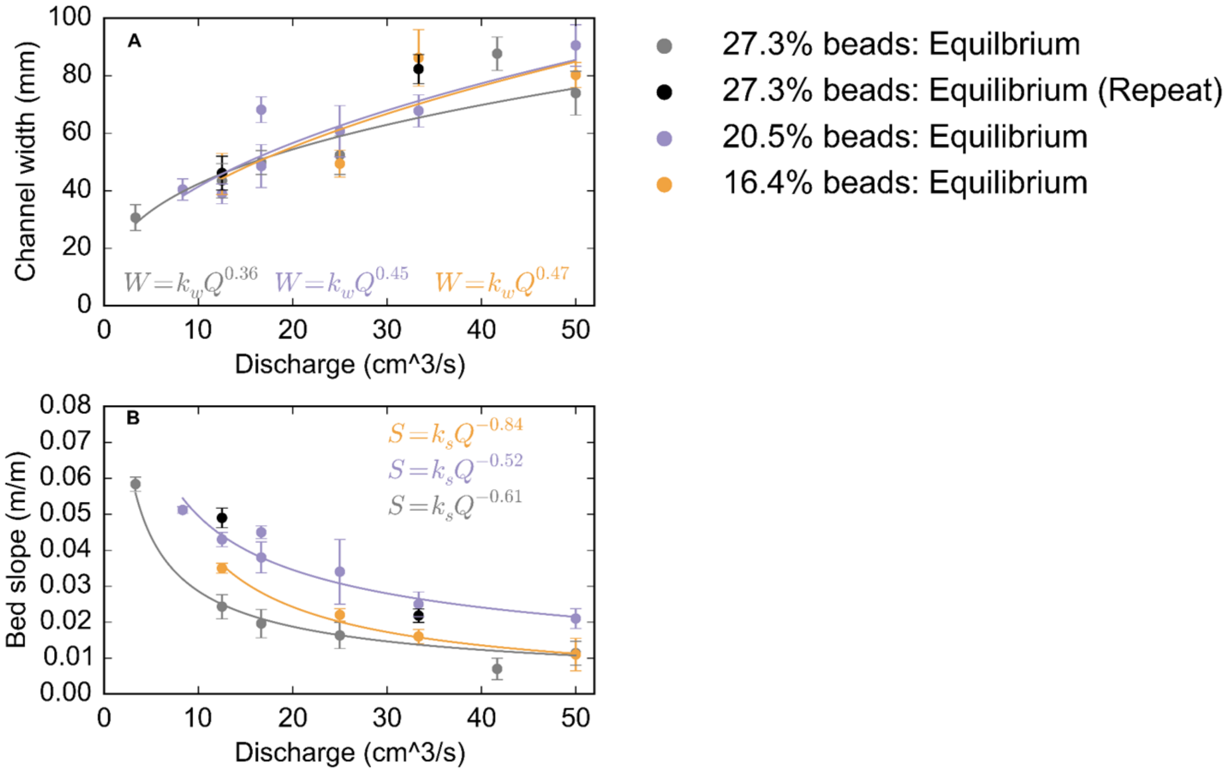

Fig. S2: Relationship between discharge and channel width (**A**) and channel bed slope (**B**) for the experimental channels. The measurements of channel width and slope were taken from the stable channel reach upstream of the knickpoint lip during the early parts of the experiment before the slope was affected by the presence of the knickpoint.

SI Section 2: Extracting hydraulic information from the experiments

To characterise the hydraulics of the experiments, the topography from the laser scanner was coupled with the *Floodos* hydrodynamic precipiton-based numerical model[6]. All point clouds in each experiment were rasterised with a pixel size of 2 mm, and *Floodos* was run using the corresponding discharge for each digital elevation model in each experiment. The flow in the experimental channels is laminar in nature with Reynolds numbers typically less than 500 (Table S1), so *Floodos* was run in the laminar-flow setting. In pure laminar flow, the friction coefficient C is entirely set by the water viscosity μ and should be approximately $C \sim \rho g/3\mu$ where ρ is water density and g is gravitational acceleration. This predicts that at 10°C, $C \sim 2.5 \times 10^6$ m^{-1}.s^{-1}, and this value has been shown to accurately match the spatial extent of water flow in experimental channels[7].

Performing *Floodos* simulations is advantageous for this data extraction, as it generates spatial masks of unit discharge and water depth, from which additional hydrological information can be calculated at the pixel scale, such as flow velocity, Froude number ($Fr = \frac{U}{\sqrt{gH}}$, where Fr is the Froude number, U is the flow velocity and H is the flow depth) and shear stress ($\tau = \rho gHS$, where τ is bed shear stress and S is the water slope (Fig. S3, Table S1). Subsequent calculations of the average value of these parameters for an entire channel mask gives the benefit of a large sample size and the removal of any manual measurement error. Additionally, channel width was extracted from the *Floodos* output using cross-sections of the wetted area for the entire length of the channel. The length of cross-sections of the wetted area for the reach of the channel unaffected by the input and output conditions (i.e. > 10 cm from each) were extracted from the initial topography (i.e., before a knickpoint was initiated through base level fall). The mean width is shown in Fig. S2, with the error bars indicating one standard deviation.

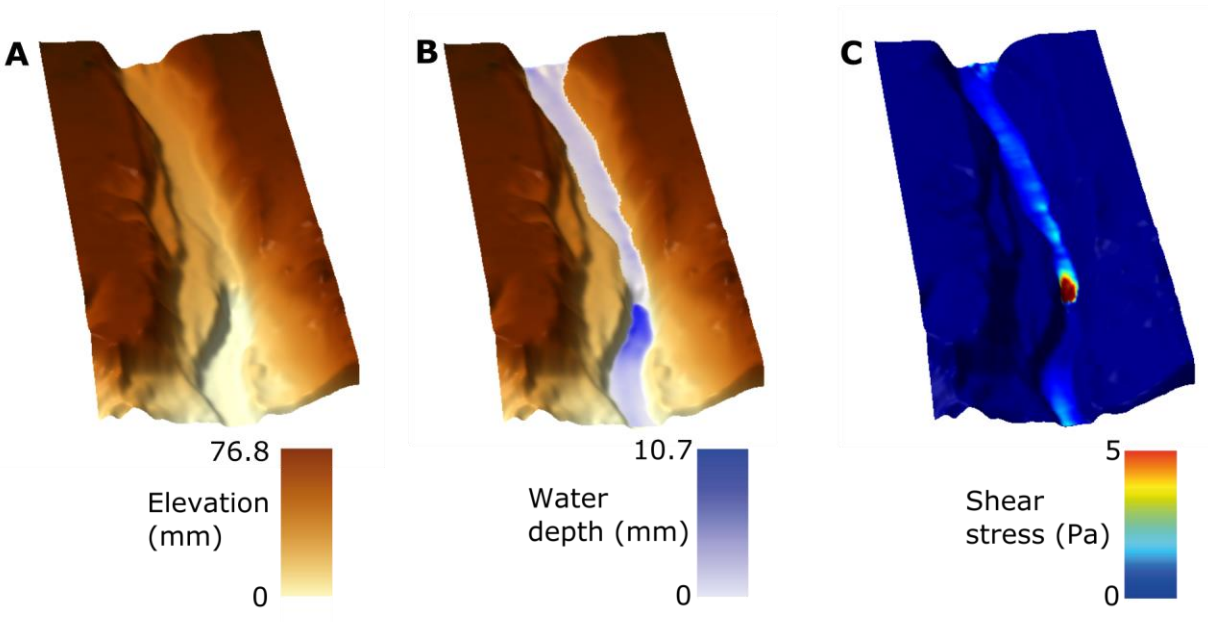

Fig. S3: Example of Floodos hydrodynamic model output for an experiment with discharge of 12.5 cm^3/s. (A) Digital Elevation Model (2 mm pixel size), generated from the laser scanner point clouds. The point clouds were generated with a green laser, allowing penetration of the water and data collection of the channel bed. (B) Mask of wetted area in the channel, coloured by the water depth. (C) Shear stress within the channel, with the highest values associated with the high slopes at the knickpoint.

SI Section 3: Experimental parameters

Table S1: Hydraulic parameters during each experiment. The water depth, flow velocity, Froude number and Reynolds number are calculated from the *Floodos* model output. Mean values are provided for all pixels not affected by the inlet or outlet (> 10 cm in distance) and uncertainty is the standard deviation. The shear stress is calculated from a mean of 20 measurements taken one channel width upstream of the knickpoint.

Date of experiment	% Mix (Angular grains : Spherical Beads : Water)	Discharge (cm^3/s)	Width of equilibrium channel (mm)	Width at KP (mm)	Slope upstream of knickpoint (m/m)	Mean water depth (mm)	Mean flow velocity in equilibrium channel (cm/s)	Mean Froude number in equilibrium channel	Mean Reynolds number in equilibrium channel	Mean Shear stress, (Pa)	Mean knickpoint retreat rate (mm/s)
Nov 2013	54.66 : 27.33 : 18	1.66	27.2 ± 4.9	37.6 ± 8.0	0.054 ± 0.021	0.69 ± 0.14	4.7 ± 1.3	0.66 ± 0.16	34 ± 13	1.92 ± 0.19	0.368 ± 0.070
Nov 2013	54.66 : 27.33 : 18	3.33	25.1 ± 4.1	25.2 ± 3.4	0.051 ± 0.005	1.03 ± 0.22	5.4 ± 2.1	0.70 ± 0.27	54 ± 28	2.20 ± 0.22	0.367 ± 0.048
Nov 2013	54.66 : 27.33 : 18	8.33	44.7 ± 6.9	21.0 ± 3.6	0.052 ± 0.011	1.56 ± 0.62	4.7 ± 1.7	0.62 ± 0.31	60 ± 19	2.64 ± 0.23	0.421 ± 0.107
Nov 2013	54.66 : 27.33 : 18	16.66	50.2 ± 5.3	18.1 ± 1.7	0.055 ± 0.009	2.86 ± 1.10	13.2 ± 5.9	1.34 ± 0.67	195 ± 85	2.62 ± 0.33	0.372 ± 0.087
Nov 2013	54.66 : 27.33 : 18	33.33	60.9 ± 6.6	41.1 ± 5.0	0.037 ± 0.006	2.26 ± 0.81	12.4 ± 4.6	1.18 ± 0.54	259 ± 98	2.86 ± 0.15	0.339 ± 0.069

Jul 2015	54.66 : 27.33 : 18	3.33	30.7 ± 4.5	0.013 ± 0.002	0.058 ± 0.002	1.10 ± 0.30	4.2 ± 1.6	0.54 ± 0.30	41 ± 20	0.8 ± 0.4	0.183 ± 0.044
Jul 2015	54.66 : 27.33 : 18	12.5	43.5 ± 6.0	0.023 ± 0.005	0.024 ± 0.003	1.77 ± 0.30	10.3 ± 4.4	1.07 ± 0.58	161 ± 68	0.53 ± 0.37	0.162 ± 0.040
Jul 2015	54.66 : 27.33 : 18	16.6	49.8 ± 4.2	0.024 ± 0.004	0.020 ± 0.004	0.94 ± 0.46	8.5 ± 3.5	1.32 ± 0.50	134 ± 64	0.29 ± 0.13	0.202 ± 0.062
Jul 2015	54.66 : 27.33 : 18	25	52.4 ± 6.7	0.028 ± 0.006	0.016 ± 0.004	2.63 ± 0.39	11.2 ± 4.4	1.11 ± 0.91	254 ± 95	0.43 ± 0.13	0.152 ± 0.064
Jul 2015	54.66 : 27.33 : 18	41.65	87.7 ± 5.8	0.037 ± 0.006	0.007 ± 0.003	4.53 ± 0.78	11.3 ± 6.9	0.99 ± 0.93	292 ± 94	0.42 ± 0.11	0.153 ± 0.055
Jul 2015	54.66 : 27.33 : 18	50	74.0 ± 7.6	0.55 ± 0.017	0.011 ± 0.003	2.36 ± 0.52	16.0 ± 6.7	1.54 ± 0.99	384 ± 147	0.53 ± 0.16	0.190 ± 0.060
Oct 2016	61.5 : 20.5 : 18	7.5	40.5 ± 3.7	25.1 ± 2.1	0.051 ± 0.001	1.36 ± 0.13	12.0 ± 2.4	1.07 ± 0.18	182 ± 62	0.42 ± 0.06	0.072
Oct 2016	61.5 : 20.5 : 18	12.5	39.0 ± 3.5	21.0 ± 3.1	0.043 ± 0.002	1.56 ± 0.10	14.2 ± 3.6	1.17 ± 0.22	266 ± 109	0.50 ± 0.07	0.089

Oct 2016	61.5 : 20.5 : 18	16.6	68.2 ± 4.5	42.8 ± 4.4	0.045 ± 0.002	1.22 ± 0.10	11.9 ± 3.7	1.09 ± 0.22	197 ± 101	0.45 ± 0.05	0.088
Oct 2016	61.5 : 20.5 : 18	16.6	48.6 ± 7.5	37.5 ± 2.6	0.038 ± 0.004	2.69 ± 1.04	12.2 ± 3.2	1.00 ± 0.24	297 ± 130	0.36 ± 0.09	0.096
Oct 2016	61.5 : 20.5 : 18	25	60.7 ± 8.9	35.1 ± 1.4	0.034 ± 0.009	2.14 ± 0.55	16.0 ± 3.4	1.23 ± 0.26	359 ± 137	0.53 ± 0.11	0.099
Oct 2016	61.5 : 20.5 : 18	33.3	67.8 ± 5.6	42.4 ± 3.4	0.025 ± 0.003	2.17 ± 0.36	17.5 ± 5.0	1.39 ± 0.42	439 ± 212	0.50 ± 0.14	0.086
Oct 2016	61.5 : 20.5 : 18	50	90.6 ± 7.2	43.8 ± 4.9	0.021 ± 0.003	3.77 ± 1.23	13.1 ± 3.4	0.88 ± 0.26	435 ± 201	0.30 ± 0.08	0.094
Nov 2017	65.6 : 16.4 : 18	12.5	45.9 ± 7.1	25.0 ± 2.8	0.035 ± 0.001	1.52 ± 0.11	13.8 ± 3.0	1.17 ± 0.27	240 ± 79	0.53 ± 0.06	0.064
Nov 2017	65.6 : 16.4 : 18	16.6	49.4 ± 4.7	26.5 ± 3.5	0.022 ± 0.002	3.01 ± 0.80	16.0 ± 4.5	1.24 ± 0.45	437 ± 184	0.45 ± 0.21	0.033
Nov 2017	65.6 : 16.4 : 18	33.3	86.2 ± 9.8	30.8 ± 3.2	0.016 ± 0.002	1.92 ± 0.12	13.7 ± 3.6	1.05 ± 0.26	336 ± 171	0.38 ± 0.07	0.066
Nov 2017	65.6 : 16.4 : 18	50	80.3 ± 4.3	35.1 ± 2.8	0.011 ± 0.005	4.58 ± 1.23	12.0 ± 2.4	0.79 ± 0.32	460 ± 178	0.25 ± 0.08	0.048
Nov 2017	54.66: 27.33 : 18	12.5	46.2 ± 5.9	1.52 ± 0.08	18.8 ± 1.8	0.049 ± 0.003	13.5 ± 2.9	1.12 ± 0.18	234 ± 84.9	0.53 ± 0.07	0.107

Nov 2017	54.66 : 27.33 : 18	33.3	82.3 ± 5.1	1.93 ± 0.08	34.4 ± 2.9	0.022 ± 0.002	15.4 ± 3.1	1.13 ± 0.20	333 ± 103	0.47 ± 0.03	0.167

SI Section 4: Relationship between discharge and bed shear stress

This section provides the derivation of the relationship between discharge and bed shear stress, allowing the interpretation of the self-organisation of channel geometry and the constant knickpoint retreat rate even under higher discharges.

The bed shear stress is calculated using the following equation:

$$\tau = \rho g H S \qquad \text{(Equation S5.1)}$$

where τ is the bed shear stress, ρ is the fluid density, g is the acceleration due to gravity, H is the flow depth (m) and S is the slope.

The discharge *Q* is calculated according to:

$$Q = VWH \qquad \text{(Equation S5.2)}$$

where V is the flow velocity, $W$is the channel width and H is the flow depth.

For low Reynold's number typical of our experimental conditions (laminar flow conditions), V can be estimated by:

$$V = CH^2 \qquad \text{(Equation S5.3a)}$$

where C is the friction coefficient[8]

Combining equations S5.2 and S5.3a gives:

$$Q = CH^3WS \qquad \text{(Equation S5.4a)}$$

Rearranging equation S5.4a, and substituting *D* into equation S5.1 gives:

$$\tau = \rho g \left(\frac{Q}{CWS}\right)^{\frac{1}{3}} S \qquad \text{(Equation S5.5a)}$$

which can be rearranged to:

$$\tau = \frac{\rho g}{C^{\frac{1}{3}}} Q^{\frac{1}{3}} W^{-\frac{1}{3}} S^{\frac{2}{3}} \qquad \text{(Equation S5.6a)}$$

From SI Section 1, we know that $W \propto Q^{\sim 0.5}$ and while the relationship between slope and discharge is not consistent between all sets of experiments (Fig. S2), the mean value is $S \propto Q^{\sim -0.5}$, similar to the typical value for the channel steepness index of 0.45 given by Whipple (2004) (ref. 5). These can be substituted into Equation S5.6a to give:

$$\tau \propto \frac{\rho g}{C^{\frac{1}{3}}} Q^{-\frac{1}{6}} \qquad \text{(Equation S5.7a)}$$

For high Reynold's number (turbulent flow conditions) typical of natural rivers, V can be estimated using the Manning's formula:

$$V = \frac{1}{n} H^{\frac{2}{3}} S^{\frac{1}{2}} \qquad \text{(Equation S5.3b)}$$

Where *n* is Manning's roughness parameter. Combining equations S5.2 and S5.3b gives:

$$Q = \frac{1}{n} H^{\frac{5}{3}} W S^{\frac{1}{2}} \qquad \text{(Equation S5.4b)}$$

Rearranging equation S5.4a and substituting *D* into equation S5.1 gives:

$$\tau = \rho g \left(\frac{nQ}{W S^{\frac{1}{2}}}\right)^{\frac{3}{5}} S \qquad \text{(Equation S5.5b)}$$

which can be rearranged[9]:

$$\tau = \rho g n\, Q^{\frac{3}{5}} W^{-1} S^{\frac{1}{2}} \qquad \text{(Equation S5.6b)}$$

Using values of $W \propto Q^{\sim 0.5}$ and $S \propto Q^{\sim -0.5}$ (e.g., Lague, 2014[10]), gives:

$$\tau \propto \rho g n\, Q^{-0.15} \qquad \text{(Equation S5.7b)}$$

Equations S5.7a and S5.7b show that there is a very weak relationship between shear stress in the channel and discharge, under both laminar and turbulent conditions, consistent with the relatively constant shear stress measured under different discharges in Fig. 4B. Under changing discharge, the channel self-organises through variations in channel width and channel slope. This self-organisation prevents higher shear stresses at the knickpoints under higher discharge. As erosion of the silica material is mainly through hydraulic shear, the constancy of shear stress here may explain why knickpoint retreat rate is not higher during the experiments with higher discharges (Fig. 3A) as predicted by the stream power incision model. This observation should hold, whether the flow is laminar (as in our experiments, see Table S1) or turbulent. The shear stress is consistent between the experiments using different silica mixes, which could explain the difference in the magnitude of the knickpoint retreat rate. For materials with different cohesions but the same shear stress exerted by the channel, the material with the stronger cohesion should erode slower when erosion is predominantly by shear.

SI Section 5: Roan Plateau, Colorado:

Data extraction and analysis for Roan Plateau

The Roan Plateau study area was selected because of the sparse vegetation cover in the area, which allowed the identification of knickpoints and measurement of their width from high-resolution aerial imagery. Knickpoints within 15 catchments were located using Google Earth Pro, and the width of the channel at the knickpoint lip extracted using the measuring tool within Google Earth Pro at each of these locations. The drainage area of the channels at each of these 15 identified knickpoints was calculated using the freely available 30 m resolution Shuttle Radar Topography Mission (SRTM) Digital Elevation Model of the area in ArcGIS. Table S2 provides the detailed information from this analysis shown in Fig. S4, including knickpoint location, knickpoint width and drainage area.

Table S2: Roan Plateau knickpoint width and drainage area data (extracted using Google Earth imagery and SRTM 30 m DEM in ArcGIS, respectively).

Coordinates (UTM zone 12N)		Knickpoint width (m)	Drainage area (km^2)
725727 E	4384006 N	1.1	3.1
754177 E	4378296 N	1.2	1.5
750803 E	4378808 N	1.4	1.1
752369 E	4379007 N	1.6	3.2
744075 E	4375658 N	1.7	1.7
738954 E	4378600 N	2.3	6.2
731781 E	4382789 N	2.5	8.4
721498 E	4390934 N	2.6	9.1

744818 E	4384870 N	2.7	12.7
723920 E	4389327 N	2.9	16.8
727890 E	4387599 N	3.3	17.7
756520 E	4383474 N	3.5	47.8
743557 E	4390490 N	3.6	53.2
752654 E	4389408 N	4.2	72.6
720339 E	4390661 N	4.7	30.5

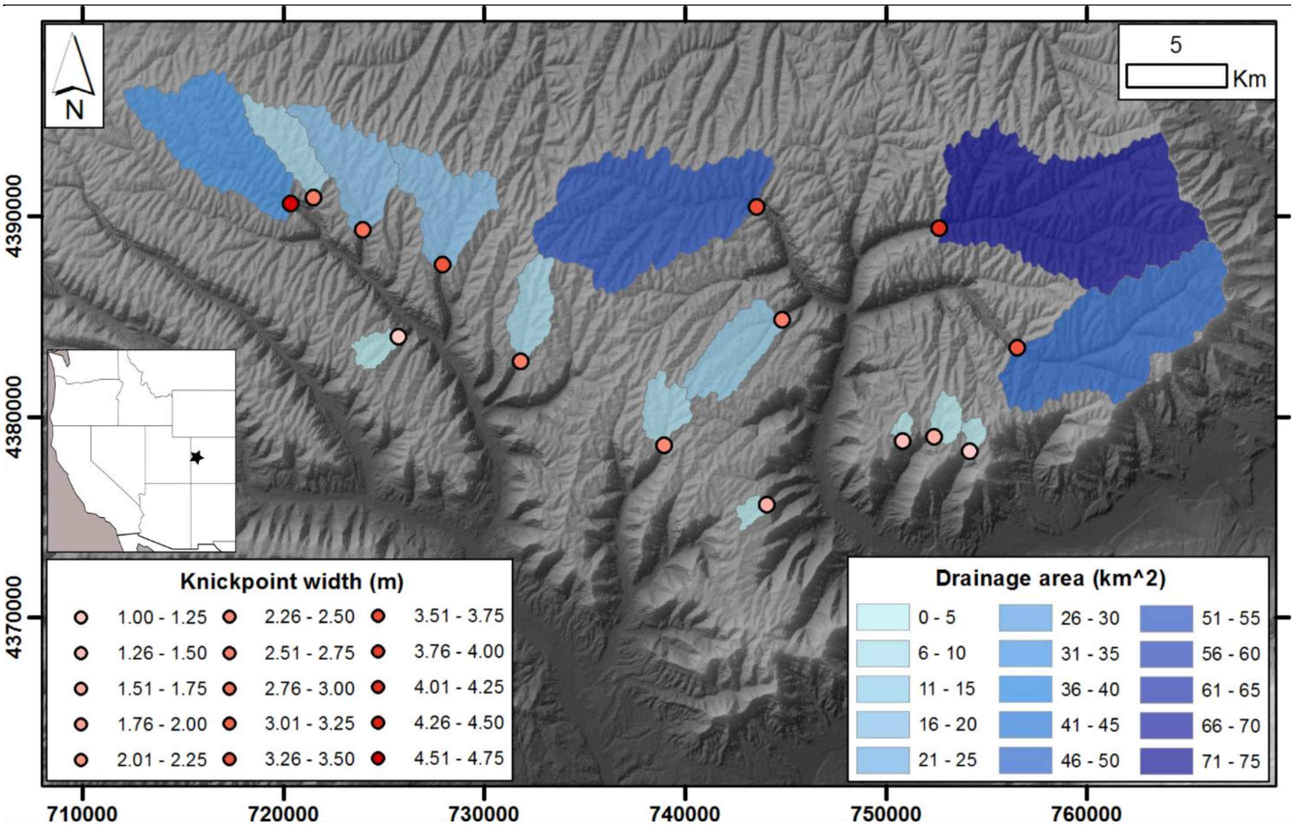

Fig. S4: Map of knickpoint width and upstream catchment drainage area data for the Roan Plateau, Colorado, USA (Inset made with Natural Earth, Free vector and raster map data @ naturalearthdata.com). Knickpoint location indicated by the points, coloured by knickpoint width, and outline of upstream drainage area indicated in blue. Coordinates are given in UTM zone 12N. Background is the 30 m resolution SRTM Digital Elevation Model overlayed onto a hillshade layer generated in ArcGIS. The influence of the knickpoints, as dynamic boundaries between a downstream area characterised by higher relief, longer and

steeper hillslopes, and an upstream area characterised by shorter and less steep hillslopes, can be clearly seen within the landscape.

SI Section 6: SI Videos

Uploaded are 2 videos showing examples of the experiments discussed in this manuscript. The videos are named according to the set of experiments that they belong to (e.g. 27.3% beads) and the discharge (e.g. 50 cm^3/s). The difference in channel width during the experiments can be clearly seen by comparing the experiment at 3.33 cm^3/s with the experiment at 50 cm^3/s.

Images were collected every 60 seconds, with the videos made with 5 images per second.

Please note that during the experiment at 3.33 cm^3/s , unfortunately, the camera mounted above the flume malfunctioned and pictures were not collected for the full duration of the experiment.

List of video files:

27percentBeads_3.33cm3s.mp4

27percentBeads_50cm3s.mp4

Supplementary Information References

1. Hooke, R.L. Model Geology: Prototype and Laboratory Streams: Discussion. *Geol Soc Am Bull* **79** (3), 391-394 (1968)
2. Lague, D., Crave, A., Davy, P. Laboratory experiments stimulating the geomorphic response to tectonic uplift. *J Geophys Res* **108** (B1), 2008 (2003)
3. Bonnet, S. Shrinking and splitting of drainage basins in orogenic landscapes from the migration of the main drainage divide. *Nature Geoscience* **2**, 766-771 (2009)
4. Crosby, B.T., Whipple, K.X. Knickpoint initiation and distribution within fluvial networks: 236 waterfalls in the Waipaoa River, North Island, New Zealand. *Geomorpholog*y **82**, 16-38 (2006)
5. Whipple, K.X. Bedrock rivers and the geomorphology of active orogens. *Annual Reviews of Earth and Planetary Sciences* **32**, 151-185 (2004)
6. Davy, P., Croissant, T., Lague, D. A precipiton method to calculate river hydrodynamics, with applications to flood prediction, landscape evolution models and braiding instabilities. *J Geophys Res: Earth Surface* **122** (8), 1491-1512 (2017)
7. Baynes, E.R.C., Lague, D., Kermarrec, J.-J. Supercritical river terraces generated by hydraulic and geomorphic interactions (in review)
8. Lajeunesse, E. *et al.* Fluvial and submarine morphodynamics of laminar and near-laminar flows: a synthesis. *Sedimentology* **57**, 1-26 (2010)
9. Howard, A.D. A detachment-limited model of drainage basin evolution. *Water Resources Research* **30**, 2261-2285 (1994)
10. Lague, D. The stream power incision model: evidence, theory and beyond. *Earth Surf Proc Landf* 39, 38-61 (2014)
